# Dengue and Dengue Virus in Guangdong, China, 1978–2017: Epidemiology, Seroprevalence, Evolution, and Policies

**DOI:** 10.3389/fmed.2022.797674

**Published:** 2022-03-21

**Authors:** Fengfu Cui, Feiwu He, Xiaorong Huang, Lina Tian, Saiqiang Li, Chumin Liang, Lilian Zeng, Huifang Lin, Juan Su, Liping Liu, Wei Zhao, Limei Sun, Lifeng Lin, Jiufeng Sun

**Affiliations:** ^1^School of Public Health, Southern Medical University, Guangzhou, China; ^2^Guangdong Provincial Center for Disease Control and Prevention, Guangzhou, China; ^3^Guangdong Provincial Institute of Public Health, Guangdong Provincial Center for Disease Control and Prevention, Guangzhou, China; ^4^School of Basic Medical Science, Guangzhou University of Chinese Medicine, Guangzhou, China

**Keywords:** dengue, dengue virus, seroprevalence, policies, molecular epidemiology

## Abstract

**Background:**

Guangdong is a hyperepidemic area of dengue, which has over 0.72 million cumulative cases within the last four decades, accounting for more than 90% of cases in China. The local epidemic of dengue in Guangdong is suspected to be triggered by imported cases and results in consequent seasonal transmission. However, the comprehensive epidemiological characteristics of dengue in Guangdong are still unclear.

**Methods:**

The epidemiology, seroprevalence, molecular evolution of dengue virus, and the development of policies and strategies on the prevention and control of dengue were analyzed in Guangdong, China from 1978 to 2017.

**Findings:**

Seasonal transmission of dengue virus in Guangdong, China was mainly sustained from July to October of each year. August to September was the highest risk period of local dengue outbreaks. Most of the dengue cases in Guangdong were young and middle-aged adults. Five hundred and three fatal cases were recorded, which declined within the last two decades (*n* = 10). The serological test of healthy donors' serum samples showed a positive rate of 5.77%. Dengue virus 1–4 (DENV 1–4) was detected in Guangdong from 1978 to 2017. DENV 1 was the dominant serotype of dengue outbreaks from 1978 to 2017, with an increasing tendency of DENV 2 since 2010. Local outbreaks of DENV 3 were rare. DENV 4 was only encountered in imported cases in Guangdong, China. The imported cases were the main source of outbreaks of DENV 1–2. Early detection, management of dengue cases, and precise vector control were the key strategies for local dengue prevention and control in Guangdong, China.

**Interpretation:**

Dengue has not become an endemic arboviral disease in Guangdong, China. Early detection, case management, and implementation of precise control strategies are key findings for preventing local dengue transmission, which may serve for countries still struggling to combat imported dengue in the west pacific areas.

## Introduction

Dengue is an arbovirus disease caused by dengue virus (DENV), which is mainly transmitted by *Aedes aegypti* or *Ae. albopictus* (1). DENV belongs to the genus *Flavivirus* of the family *Flaviviridae*, and could be divided into four distinct serotypes (DENV 1–4) according to its antigenicity (1). Nearly half (47.7%) of the dengue outbreaks from 1990 to 2015 had more than one DENV serotype coepidemic. After 2010, DENV 1 and DENV 2 were the most epidemic in Africa, America, and Europe. DENV 1 was predominant in the Western Pacific region, while DENV 2 and DENV 3 were most frequently detected in the Eastern Mediterranean region (2). DENV 4 showed consistent increase in Asia and the Americas (3). The genome size of DENV is about 11.7 kb, encoding 3 structural proteins and 7 non-structural proteins (4). The clinical manifestations of infecting DENV vary from mild to severe which can be asymptomatic infection, fever, dengue hemorrhagic fever (DHF), and dengue shock syndrome (DSS) (5). The crossprotection of antibodies to DENV can last for a few months (6), while the secondary infection with divergent serotypes may lead to DHF and DSS (7). The current strategies of dengue prevention and control still focus on the vector control and management of cases due to the nonavailability of vaccines or effective clinical therapy protocols (8, 9).

The incidence of dengue has increased by 30-fold over the past 50 years (10). WHO declared that about 2.5 billion individuals were under threat of dengue, and over 100 million people suffered from DENV infection annually (10). Moreover, about 0.5 to 1 million people developed DHF/DSS, which eventually led to about 25,000 deaths. Dengue has become one of the most serious global public health concerns (11). Bhatt et al. (12) estimated that about 390 million people were infected with DENV each year, including 96 million symptomatic patients accounting for close to 25% of the total case number. The Asia Pacific region is the most severe area of DENV transmission currently, with more than 75% of the population living at the risk of DENV infection (12). The hospitalization rate of dengue in children had reached a staggering 19% in the Asia region (13). Which has become a serious economic and medical resource burden for those developing countries in Southeast Asia.

Southeast China is a hyperepidemic region of dengue. Outbreaks of dengue were almost reported annually in Guangdong, Guangxi, Fujian, and Yunnan provinces of this region (14). The earliest record of a dengue outbreak was in 1978 in the Guangdong province (9). Three dengue epidemics outbreak occurred in Guangdong in 1980 (4,54,205 cases), 1986 (1,18,987 cases), and 2014 (45,236 cases), respectively (15, 16). Although sporadic outbreaks of dengue were also reported in other provinces, e.g. Hainan, Zhejiang, and Henan in China (14), Guangdong ranked at the top as the most hyperepidemic area of dengue in China. During the last four decades since then, over 0.72 million dengue cases were reported in Guangdong province accounting for more than 90% of total archived dengue cases in China (17). The guidelines for dengue prevention and control have referred to the guidelines issued by WHO and adapted according to the practical experiences of Guangdong province. These guidelines were first issued in 1979 and updated in 1987 and 2015, respectively. However, the comprehensive epidemiological characteristics of dengue in Guangdong, China are still unclear, e.g whether dengue has become a localized arbovirus disease in this area? What are the demographic, seasonal, or clinical trends of dengue in those populations? How do the local authorities develop their strategies against dengue? Although a few studies focusing on partial or single hospital data stated potential trends (2, 9, 14–17), a study to address these remaining questions in a comprehensive way is still needed. In this study, based on the epidemiological data from the National Notifiable Infectious Disease Reporting Information System in China, archived laboratory data and policy documents of Guangdong Provincial CDC, serological testing, and DENV sequencing data, our goals were as follows: (1) describe the epidemiological characteristics of dengue in Guangdong, China by comparing that with dengue endemic areas in Southeast Asian countries, (2) explore characteristics of DENV antibodies presented in sera samples from Guangdong healthy populations through regional serological investigations, (3) draw the evolution history of four serotypes of DENV by using a comprehensive local and international DENV Envelope gene data set. Finally, we summarized the development history of guidelines against dengue in Guangdong, China. The data presented in this study will be a window for understanding the characteristics of dengue in Guangdong, China, and may serve as a model for countries that are still struggling to combat imported dengue in west pacific areas.

## Methods

### Data Collection

Dengue surveillance data during the period of 1978–1989 were obtained from local authorities records archived (hard copy data, request with permission) in Guangdong Provincial Center for Disease Control and Prevention (Guangdong CDC). Dengue was recognized as a National Notifiable Infectious Disease in China in 1989 and was diagnosed in accordance with the unified criteria published by the Chinese Ministry of Health. From 1990 to 2004, dengue was reported monthly in all provinces in China (electronic data, request with permission). During 2005–2017, dengue was reported by sentinel hospitals within 24 h of diagnosis to the National Notifiable Infectious Disease Reporting Information System (online database). The online data set charts containing the gender, age, imported or local cases, and the onset date were collected accordingly. The duplicate records from the same cases identified with personal ID were deleted.

### Definition of Dengue Case

Dengue was diagnosed based on the unified criteria issued by the National Health Commission of the People's Republic of China. Cases presented in this study were diagnosed according to the former three versions (16) (Supplementary File 1).

### Serum Sample Collection

For dengue cases and healthy donor's blood sampling, we used sterile vacuum drying tubes to collect the non-anticoagulated blood and separated the serum within 2 h after collection. The serum was placed in an internal thread cryogenic vial with a gasket inside and stored at −70°C until use for antibody screening. For dengue cases, serum samples were collected in sentinel hospitals and delivered through the sample-transfer system of Guangdong CDC. Virus detection and isolation were conducted in the laboratory of Guangdong CDC. For crosssectional serological surveys in healthy donors, the serum samples were taken from our previous nutrition survey project implemented in 14/21 counties of Guangdong in 2015 using a multiple-stage cluster random sampling approach (residents, without profession limit) (18). The serum samples were collected in community hospitals, delivered through same sample transfer system of Guangdong CDC, and stored at −70°C until use for antibody screening. A total of 1,801 serum samples were randomly selected from 8,610 serum samples to test the presence of dengue IgG/M antibodies.

### Dengue IgG/IgM Antibodies Detection

Dengue IgG and IgM antibodies to DENV 1–4 were detected by enzyme-linked immunosorbent assay (ELISA) using IgG/IgM ELISA commercial kits (19). The tests were conducted according to the manufacturer's instructions (Supplementary File 1) in the laboratory of Guangdong CDC.

### Laboratory Test and Isolation of DENV

Viral RNA in serum samples were extracted using the QIAamp Viral RNA Mini Kit (Qiagen, Hilden, Germany) (20). DENV was detected by a commercial DENV real-time quantitative RT-PCR kit (DAAN Gene, China) with the primers as previously reported (20). Dengue virus was isolated by C6/36 cells inoculation. C6/36 cells were cultured under 28°C and the serum samples were added and adsorbed for 1 h respectively. After every three days, the cells were checked for cytopathic effect (CPE). If there was no CPE after the first 7 days, culture supernatants were transferred to new C6/36 cells for another 7 days of observation and repeated a third time if the second culture period also resulted in no CPE. Serum samples showing no CPE after three consecutive 7-day culturing periods were considered negative for DENV. DENV identification was performed using the same real-time RT-PCR kit as above.

### DENV E Gene Amplification and Sequencing

Once the isolate was confirmed as DENV, one-step RT-PCR method (Qiagen, Germany) was used to amplify the DENV E gene sequences. The PCR product was checked by agarose gel electrophoresis analysis, and the positive product was purified by the QIA PCR rapid product purification kit (Qiagen, Germany). The purified product was sequenced by ABI PRISMH 3700 (Applied Biosystems, Shanghai, China). The homology analysis was conducted by online alignment software in GenBank (www.blast.ncbi.nlm.nih.gov/Blast.cgi).

### Evolution of DENV

To obtain the DENV reference sequences, we searched for all the nucleotide sequences of DENV by using “Dengue virus” as the search term in GenBank. Sequences were divided into four FASTA files named DENV 1–4 and aligned in the Mega 6.06 program (21). The sequences without detailed information, e.g sampling time and location, were removed from the data set. Bioedit software was used to check the homology similarity for the above preliminary screened sequences. If the homology was more than 99% for any potential outbreak data set, only one representative sequence was randomly retained in the same country (region) and the same year. According to the same principle of sequence selection as the reference international sequences, only one sequence with more than 99% homology in the same year was randomly selected.

Maximum likelihood evolutionary trees of combination between four types of DENV data were constructed by Mega6.06 software with GTR+Gamma model (21), and the sequences whose genetic distance was inconsistent with the time of isolation were removed after verification. TempEst was used to evaluate the genetic distance and time distribution (22). BEAST v.1.10.4 was used to perform Bayesian phylogenetic inference analysis of DENV. The nucleic acid replacement model was set as GTR+G (23). Relaxed uncorrelated molecular clock was used as the molecular clock model. Three independent Markov chains Monte Carlo (MCM) runs of 100 million steps were computed for DENV (10% burn-in). Convergence (ESS > 200) and behavior of the MCM were inspected using Tracer v1.6 (http://beast.bio.ed.ac.uk/Tracer). The summary phylogenies were visualized in FigTree v.1.4.2 (http://tree.bio.ed.ac.uk/software) and annotated by iTOL (https://itol.embl.de/itol.cgi).

### Reforming of Control Strategies

The policies for the prevention and control of dengue in Guangdong province from 1978 to 2017 were collected from local public health authorities archived in Guangdong CDC.

### Statistical Analysis

The average level of the incidence of dengue within the last four decades was presented by mean and 95% confidence interval. The analysis for the effect of age, sex, and their interaction on the annual incidence of dengue was performed by applying two-way classification ANOVA. The correlation between the local and imported cases was assessed by Pearson's correlation coefficient. Differences in IgG positivity rates between sampled cities were compared by age and gender using the Chi-square test and Fisher's exact test. Time series of each year between 1990 and 2017 was analyzed to determine the heterogeneity of dengue epidemic tendency by using a Meta approach. The level of significance (α) was 0.05. R statistical software (R Foundation for Statistical Computing, V3.6.1, Vienna, Austria) was employed to produce graphs, heat maps, and plots of geographical distribution.

## Results

### Epidemiology Findings of Dengue in Guangdong: 1978–2017

**Figure 1:**
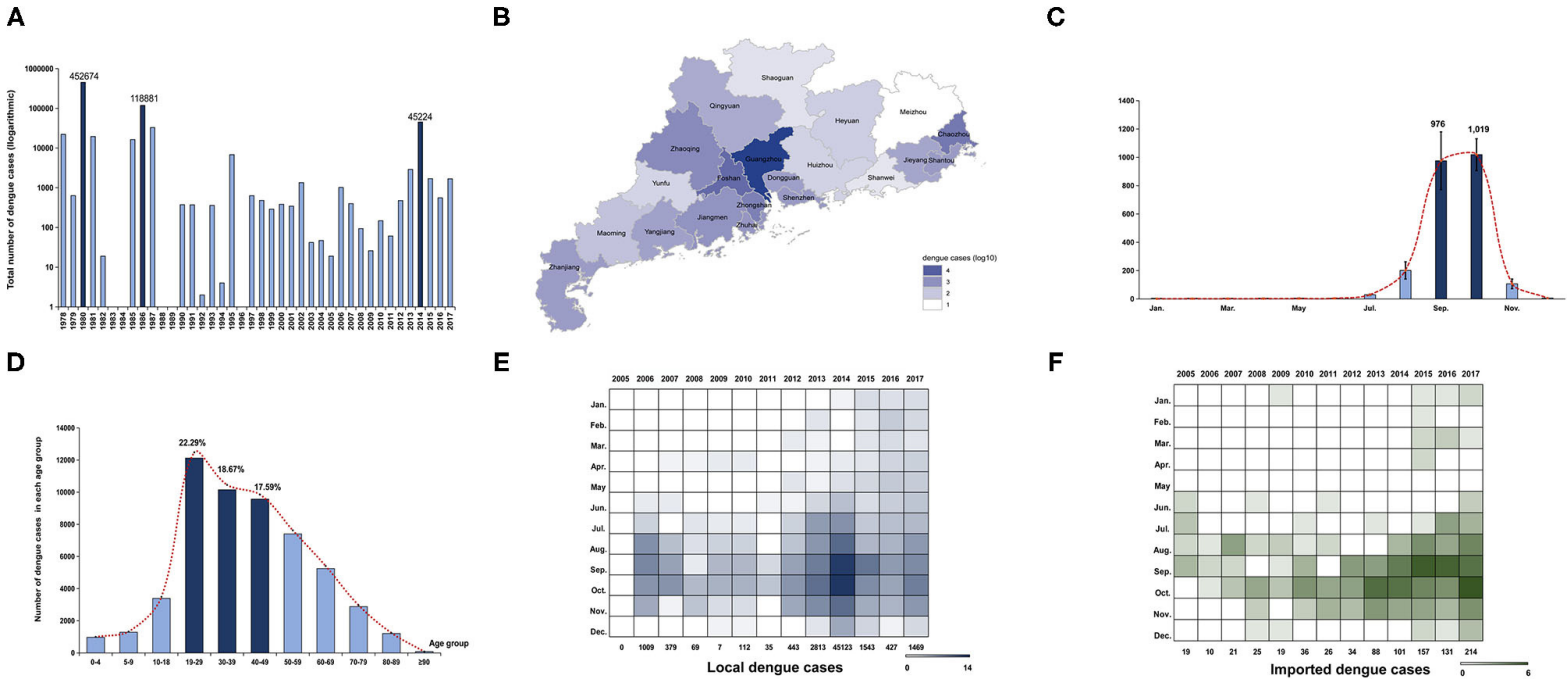
Epidemiology characteristics of dengue in Guangdong from 1978 to 2017. **(A)** Annual number of dengue cases in Guangdong from 1978 to 2017. The *Y* axis is displayed on the logarithmic scale (log10). Three major dengue epidemics are marked by dark blue. **(B)** Geographic distribution of accumulated dengue cases from 1990 to 2017 in 21 cities of Guangdong. The number of dengue cases in each city is displayed by colors of shades according to logarithmic form (log10). **(C)** Seasonal distribution of dengue in Guangdong from 1990 to 2017. The number of cases in each month is presented by the average number within the last 28 years. The incidence peak is shaded according to logarithmic scale (log10). **(D)** The age distribution is presented by accumulated dengue cases from 2005 to 2017 in each age group. The more affected groups are marked by dark blue. **(E,F)** The number of local and imported cases each month is shaded according to the logarithmic scale (log2).

A total of 729,036 dengue cases was reported in Guangdong province during 1978–2017, with 503 deaths. The incidence rate of dengue was 23.15/million within the last four decades [95% confidence interval (−5.03, 51.33)]. Three historical epidemic dengue outbreaks occurred in 1980 (452,674 cases; 7,899.36/million), 1986 (118,881 cases; 1,887.09/million), and 2014 (45,189 cases; 421.71/million) (Figure 1A). Dengue incidence varied substantially from year to year. During 1978–1989, six high-intensity outbreaks of dengue occurred. Two dengue epidemics (1980, 1986) account for 78.4% of all cases, while the intensity of outbreaks declined after 1989 when dengue became a National Notifiable Infectious Disease in China. Moderate outbreaks were encountered between 1989 and 2017, except for 1996 and 2014. Unexpectedly, there were several report gaps of dengue in 1982, 1983, 1984, 1988, 1989, 1992, 1994, and 1996.

To avoid the data bias caused by report gaps and outbreaks in 1980 (452,674 cases,62.1%) and 1986 (118,881 cases,16.3%), the geographical and seasonal distribution of dengue cases was described separately, from 1978 to 1989 and 1990 to 2017. The cumulative number of dengue cases in 21 cities of Guangdong from 1978 to 1989 showed that Foshan and Zhanjiang were the two cities initially most affected by dengue, followed by Guangzhou (Supplementary Figure 1A). During 1990–2017, Guangzhou, the capital of Guangdong, was the epicenter of dengue, followed by Foshan and Chaozhou (Figure 1B). It was worth noting that the distribution of cases has expanded to all 21 cities in Guangdong, and the epicenters of dengue have expanded to Chaozhou, signaling increased risk and the need for local prevention and control in those remote cities from Guangzhou.

A monthly analysis of dengue cases in Guangdong from 1978 to 1989 showed that outbreaks of dengue mainly occurred from June to October of each year (*n* = 545,204, 82.22%) and reached the peak level in August and September (Supplementary Figure 1B). From 1990 to 2017, most of the cases were concentrated in July to November (*n* = 65,306, 99.25%), and reached the peak level in September and October (Figure 1C). Additional analysis of each year between 1990 and 2017 indicated a consistency of dengue outbreaks from July to November, with a few years showing disease activity in the other months (Supplementary Figure 2A). Metaanalysis of yearly peak period found that the overall peak time was September—October, with medium heterogeneity (I^2^ = 67.90%, *p* < 0.01) (Supplementary Figure 2B).

Due to the lack of detailed information on dengue cases before 2005 (the online reporting system of dengue cases was not established yet), a 13-year data set of 54,310 cases from 2005 to 2017 was used to describe the demographic characteristics of dengue cases in Guangdong (Figure 1D). Dengue was reported in all age groups, mainly in young and middle-aged adults. A total of 39,249 cases were middle-aged and young people aged 19–59, accounting for 72.16% of all the reported cases. Among them, cases aged 0–18 only accounted for 10.38% (*n* = 5,644) and cases aged 19–39 accounted for 40.96% (*n* = 22,276). No gender bias was detected (male, 27,002, 49.72%; female, 27,308, 50.28%). Gender showed no significant influence on the annual incidence of dengue (F_[1,54298]_ = 0.080, *p* > 0.05). However, age showed a significant influence on the incidence of dengue (F_[5,54298]_ = 7.144, *p* < 0.001). The further interaction analysis between gender and age showed no influence with each other (F_[5,54298]_ = 1.269, *p* > 0.05).

A comparison analysis between the imported and local cases in Guangdong from 2005 to 2017 showed that the number of imported cases increased from 19 to 214 during 2005–2017 (Figures 1E,F). The distribution of imported cases (*n* = 881) showed similar trends with local cases (*n* = 53,429) in the same period (*r* = 0.335, *p* < 0.001). After 2013, imported cases in Guangdong were detected in almost every month of the year. Imported cases mainly came from Southeast Asian countries (*n* = 259, 78.01%), e.g. Thailand (*n* = 75, 22.59%), Malaysia (*n* = 48, 14.46%), and Indonesia (*n* = 44, 13.25%).

### Antibodies in a Healthy Population

**Figure 2:**
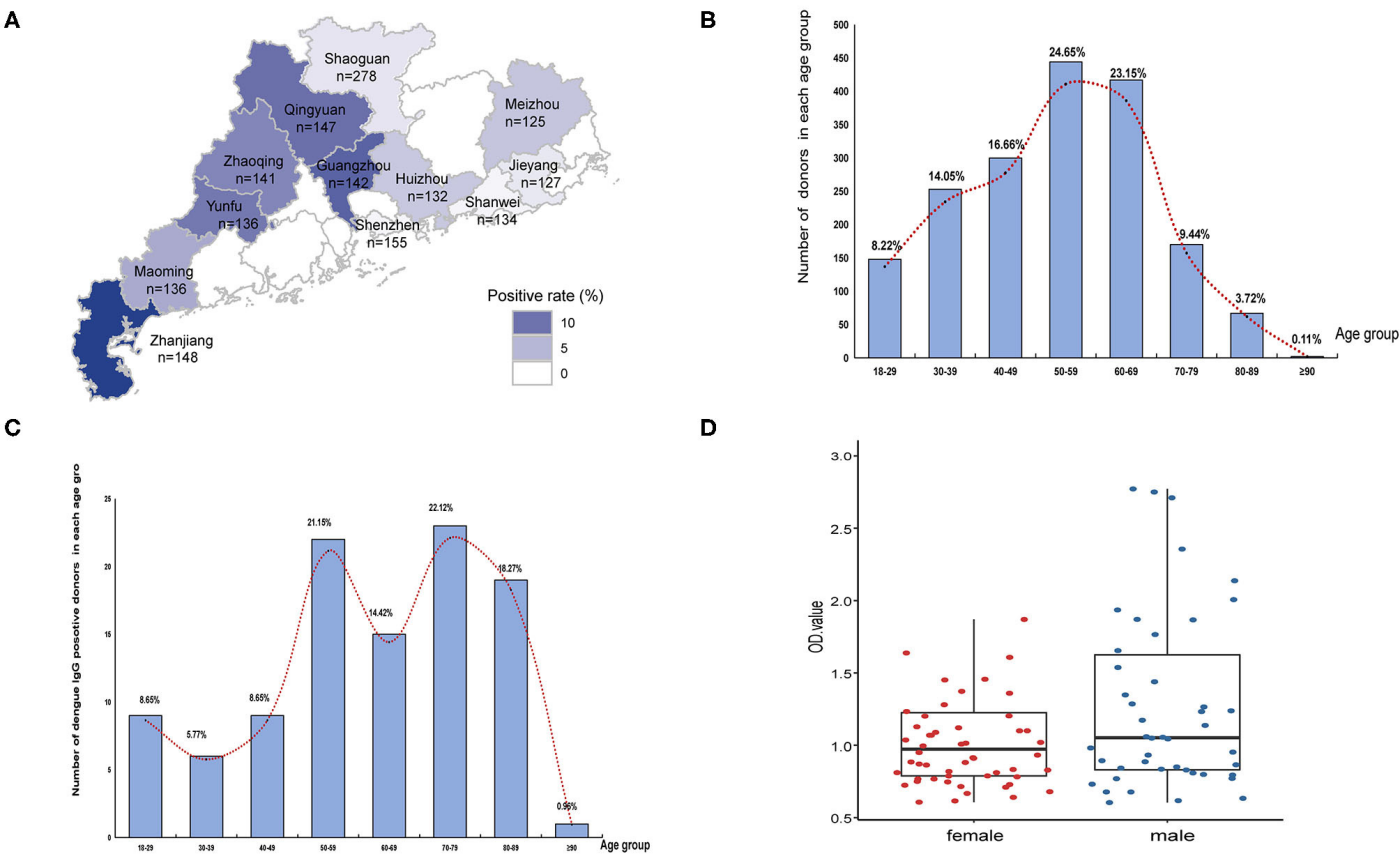
Seropravelence of dengue in Guangdong. **(A)** Geographic distribution of dengue IgG antibodies positivity rate in 11 cities of Guangdong. The areas with high positivity rates are marked by dark blue. **(B)** Age distribution of all donors (*n* = 1,801). **(C)** Age distribution of seropositive donors (*n* = 104). The distribution is presented by the number of positive donors in each age group. **(D)** Distribution of seropositive ELISA test data in female and male.

Among 1,801 randomly selected serum samples, 104 samples were positive for dengue IgG but not IgM antibodies. A positive rate of 5.77% was consistent with previous monitoring data of healthy population in Guangdong province (Figure 2). The geographical seroprevalence analysis showed that the seropositivity rates of Zhanjiang (14.19%), Guangzhou (11.27%), and Qingyuan (10.20%) were higher than those in other cities (Figure 2A) (X2= 65.55, *p* < 0.001), suggesting that further vector surveillance and serum screening should be conducted in particular remote cities, e.g. Qingyuan, a city with a rare report of dengue historically. Age had a significant influence on IgG positivity rates (X2 = 118.86, *p* < 0.001), and the age distribution of seropositive donors showed a broad peak among middle-aged and elderly adults (ages 50–89) (Figures 2B,C). In contrast, gender had no effect on IgG ELISA outcomes (X2 <0.001, *p* > 0.05).

### Evolution of DENV in Guangdong From 1978 to 2017

A total of 8,391 DENV E gene sequences with clear source and sampling information were screened in GenBank. After filtration by established screening categories, 1,575 international reference and 431 local DENV E gene sequences were included for further combined evolution analysis. The preliminary reconstruction of phylogeny analysis indicated that 431 local sequences were classified into DENV 1 (*n* = 279), DENV 2 (*n* = 75), DENV 3 (*n* = 61), and DENV 4 (*n* = 16). Genotype I of DENV 1 was the leading genotype of outbreaks in Guangdong during 2004–2017 (except for 2014 with genotype V). Cosmopolitan genotype was the dominant genotype of DENV 2 after 2010 in Guangdong. Local outbreaks of DENV 3 were rare. DENV 4 was only encountered in imported cases in Guangdong, China.

### DENV 1

**Figure 3:**
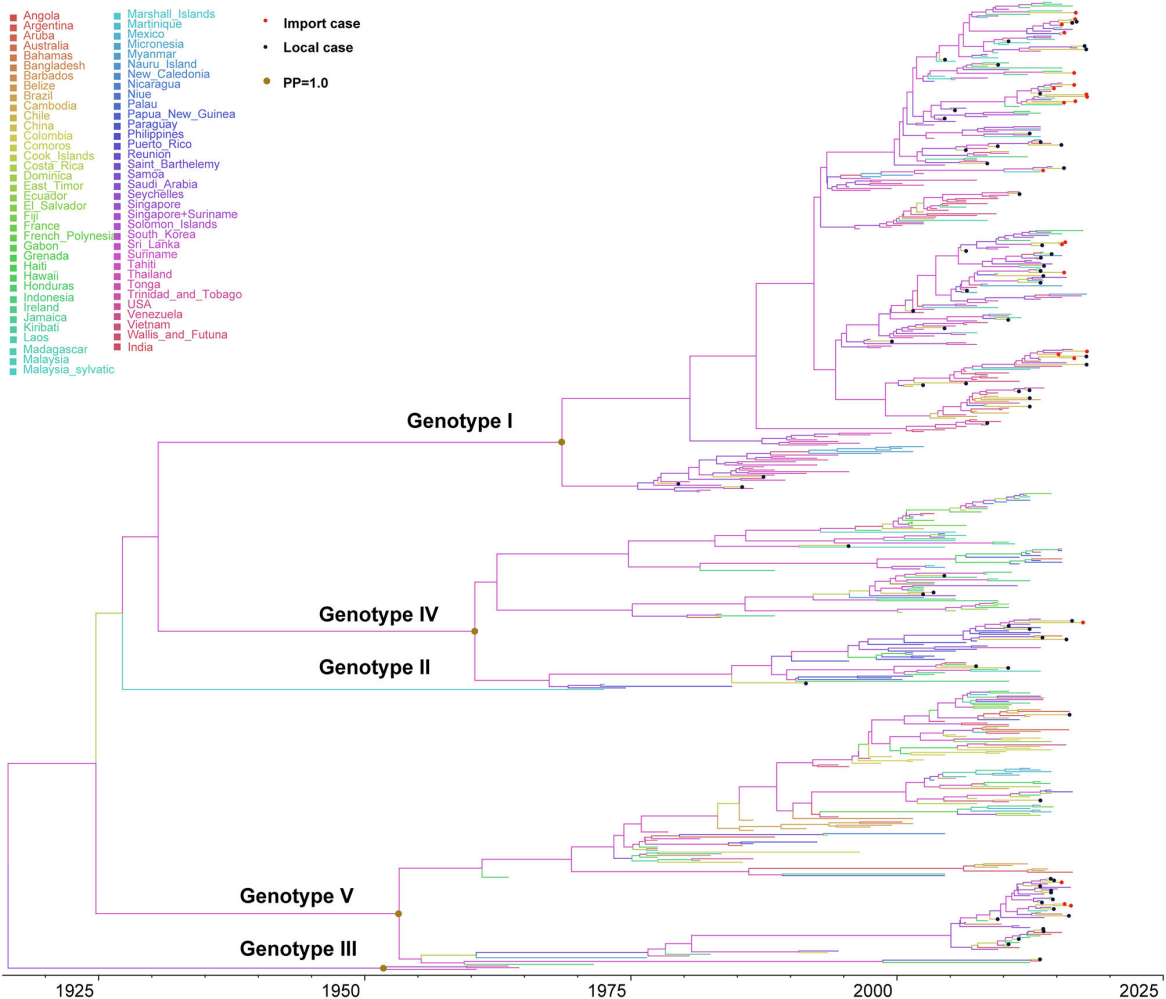
Bayesian evolutionary tree of DENV 1. Source countries/regions of each strain and different genotypes of DENV 1 are presented by colors. Red dots represent imported cases, blue dots represent local cases, and brown dots are internal branch points with posterior probability = 1.0.

DENV 1 contained five major genotypes (genotype I–V), of which genotype I, IV, and V were the primary epidemic genotypes. Genotype II and III were extinction clades (Figure 3). The inferred origin time of DENV 1 was 1916 (95% highest posterior density (HPD) and 1901–1931), while origin times for genotypes I, IV, and V were estimated as 1968 (95% HPD, 1963–1974), 1960 (95% HPD, 1955–1966), and 1953 (95% HPD, 1948–1959), respectively. The overall evolution rate of DENV 1 was 7.725 × 10^−4^ nucleotides/site/year (95% HPD, 6.998 × 10^−4^-8.429 × 10^−4^).

Genotype I primarily circulated in Southeast Asian countries (Supplementary Figure 3). The evolution rate of genotype I was 8.672 × 10^−4^ nucleotides/site/year (95% HPD, 7.807 × 10^−4^-9.530 × 10^−4^), which is slightly larger than the evolution rate of DENV 1, but there was no significant difference (*p* > 0.05). The reconstructed phylogenetic tree of genotype I showed that it can be divided into different subclusters. The strains exported to China were from different subclusters, with a continuous increase since 2000. Moreover, we found short-term local transmission events with imported cases, specifically in the subcluster of Malaysia–Indonesia–Singapore. The only doubtful cluster is the third branch on the Malaysia-Indonesia-Singapore sub-cluster (Supplementary Figure 4). A separate evolutionary analysis found that the evolutionary rate of this branch was 1.038 × 10^−3^ nucleotides/site/year (95%HPD, 7.869 × 10^−4^-1.296 × 10^−3^), higher than the evolution rate of genotype I, but it is still within the same confidence interval (Supplementary Figure 5). We found this branch was initially imported into China in 2006, and then reemerged in Guangzhou, Foshan, and Zhongshan in 2009, 2012, and 2015, while we also found sequences of strains in Singapore and Australia.

Genotype IV contained three epidemic clusters (Supplementary Figure 5), one was mainly prevalent in Philippines, another circulated in Indonesia, and the third branch was mainly epidemic in the South Pacific and South America. The differentiation time of genotype IV was about 1959, and the evolution rate was 9.051 × 10^−4^ nucleotides/site/year (95% HPD, 8.123 ×10^−4^-1.002 × 10^−3^). Genotype IV strains from China were few and scattered, and there were no apparent aggregation branches, suggesting that they were only sporadically imported into China.

Genotype V contained three clusters (Supplementary Figure 6), one of which was epidemic in South America, and the other was prevalent in Singapore and Indonesia. The third branch mainly circulated in Africa. The origin and differentiation time of genotype V was about 1955 (95% HPD, 1954–1955), and the evolution rate was 7.526 × 10^−4^ nucleotides/site/year (95% HPD, 6.798 × 10^−4^-8.238 × 10^−4^). The strains found in China mainly came from the Southeast Asian clusters, and there were only a few imported strains in the American and African clusters. The strains of the Asian clusters can be divided into two subclusters, both of which were transmitted from India to Singapore, and then finally into China. The strain of the first subclusters was the major epidemic cluster causing the large dengue outbreak in 2014. Subsequently, it was also found in Guangdong, Anhui, and other provinces of China in 2015.

### DENV 2

**Figure 4:**
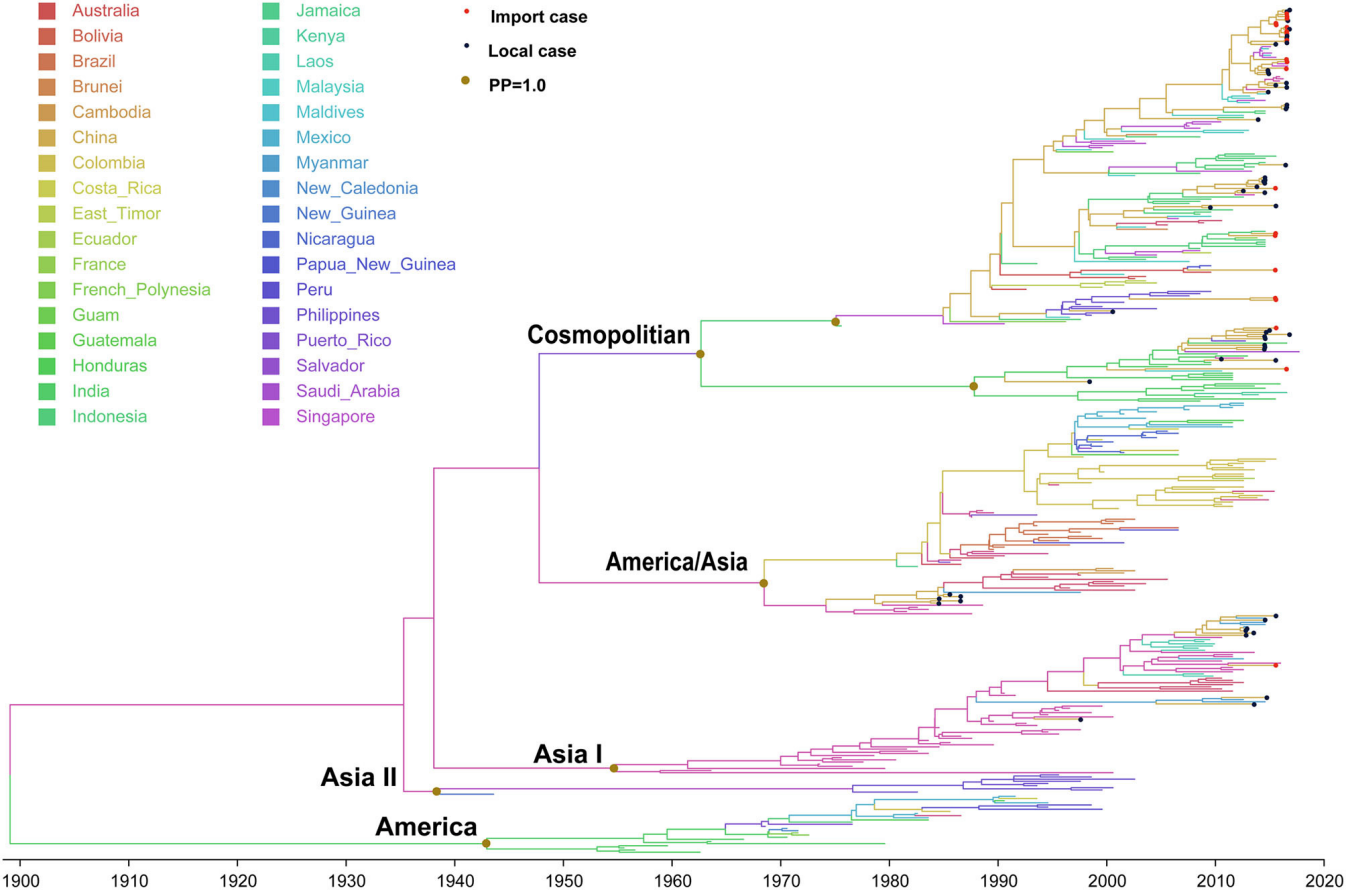
Bayesian evolutionary tree of DENV 2. Source countries/regions of each strain and different genotypes of DENV 2 are presented by colors. Red dots represent imported cases, blue dots represent local cases, and brown dots are internal branch points with posterior probability = 1.0.

The evolutionary tree of DENV 2 showed that this serotype contained five main genotypes (genotype cosmopolitan, America/Asia, Asia I, Asia II, and America) (Figure 4), among which genotype cosmopolitan, America/Asia, and Asia I were the main prevalent genotypes. Genotype Asia II and America were both early discovered strains that were limited to Philippines and South America, respectively. The origin of DENV 2 was presumed to be 1898 (95% HPD, 1882–1915), and the origin time of genotype cosmopolitan, America/Asia, and Asia I were presumed to be 1962 (95% HPD, 1956–1969), 1968 (95% HPD, 1963–1969), and 1954 (95% HPD, 1950–1956), respectively. The variation frequency of DENV 2 was 8.338 × 10^−4^ nucleotides/site/year (95% HPD, 7.671 × 10^−4^-9.001 × 10^−4^).

Genotype cosmopolitan was the major epidemic genotype of DENV 2. It could be divided into two clusters. One was mainly prevalent in Indonesia, Malaysia, and Singapore of Southeast Asia, and another one circulated in India. The divergence time was about 1947 (95% HPD, 1947–1954), which was similar to the time when genotype V in DENV 1 spread from Thailand to India and Indonesia. A majority of the DENV 2 found in China came from this genotype. Both clusters were reported in China, and also the causative strain dengue outbreak in Chaozhou, Guangdong in 2015.

Genotype America/Asia contained two clusters of America and Asia. The divergence time of the two clusters is about 1968 (95% HPD, 1961–1968). We only detected some strains in the Asia cluster around 1987, and no imported or local outbreak strains were detected since then. Meanwhile, no outbreak isolates were found in the Asia cluster of Genotype America/Asia after 2010, which means it had become an extinction cluster with no further transmission.

Genotype Asia I originated in Thailand (1954, 95% HPD, 1949–1963), then expanded to Vietnam, Myanmar, and Laos. It began to be exported into Yunnan, China in 1998 and 2006.

### DENV 3

DENV 3 contained five major genotypes (genotype I-V) (Supplementary Figure 7), in which genotypes I–III were the main epidemic genotype. The origin of DENV 3 was presumed to be 1898 (95% HPD, 1882–1915), and the origin of genotypes I–III were presumed to be 1978 (95% HPD, 1975–1984), 1965 (95% HPD, 1963–1969), and 1952(95% HPD, 1948–1958), respectively. The variation frequency of DENV 2 was 9.326 × 10^−4^ nucleotides/site/year (95% HPD, 8.031 × 10^−4^-1.072 × 10^−3^).

The inferred origin of genotype I was in India and Sri Lanka with four clusters, two of which were still circulating in the origin region. The third cluster had expanded to central and South America. The fourth cluster circulated in the Indian Ocean and Thailand. The strains of this genotype found in China were mainly imported from the Indian Ocean region island countries and Thailand. Genotype II originated in Thailand. The first cluster of genotype II had expanded from Thailand to Vietnam and Cambodia, and the second cluster was mainly expanded to Laos, Myanmar, and Thailand. The viruses exported to China were mainly from the second cluster, most of which had been introduced into Guangdong and Yunnan from Laos since 2007. Genotype III originated in Indonesia, and then expanded to Malaysia and Singapore, Philippines, and Haiti in Central America.

### DENV 4

DENV 4 contained 3 main genotypes I–III and a sylvatic genotype (Supplementary Figure 8). The origin time of DENV 4 was speculated to be 1900 (95% HPD, 1869–1942), with a wide range of confidence intervals, while the origin times of genotypes I–III were 1955 (95% HPD, 1948–1966), 1942 (95% HPD, 1934-1951), and 1928 (95% HPD, 1910–1947), respectively. The evolution rate of DENV 4 was 1.099 × 10^−3^ nucleotides/site/year (95% HPD, 9.481 ×10^−4^-1.248 × 10^−3^). Genotype II had been exported to Yunnan through Myanmar, Cambodia, and Laos in 2007. Only a few imported genotype II cases were detected in Guangdong, while no local outbreaks occurred.

### Reforming of Control Strategies From 1978 to 2017 in Guangdong

**Figure 5:**
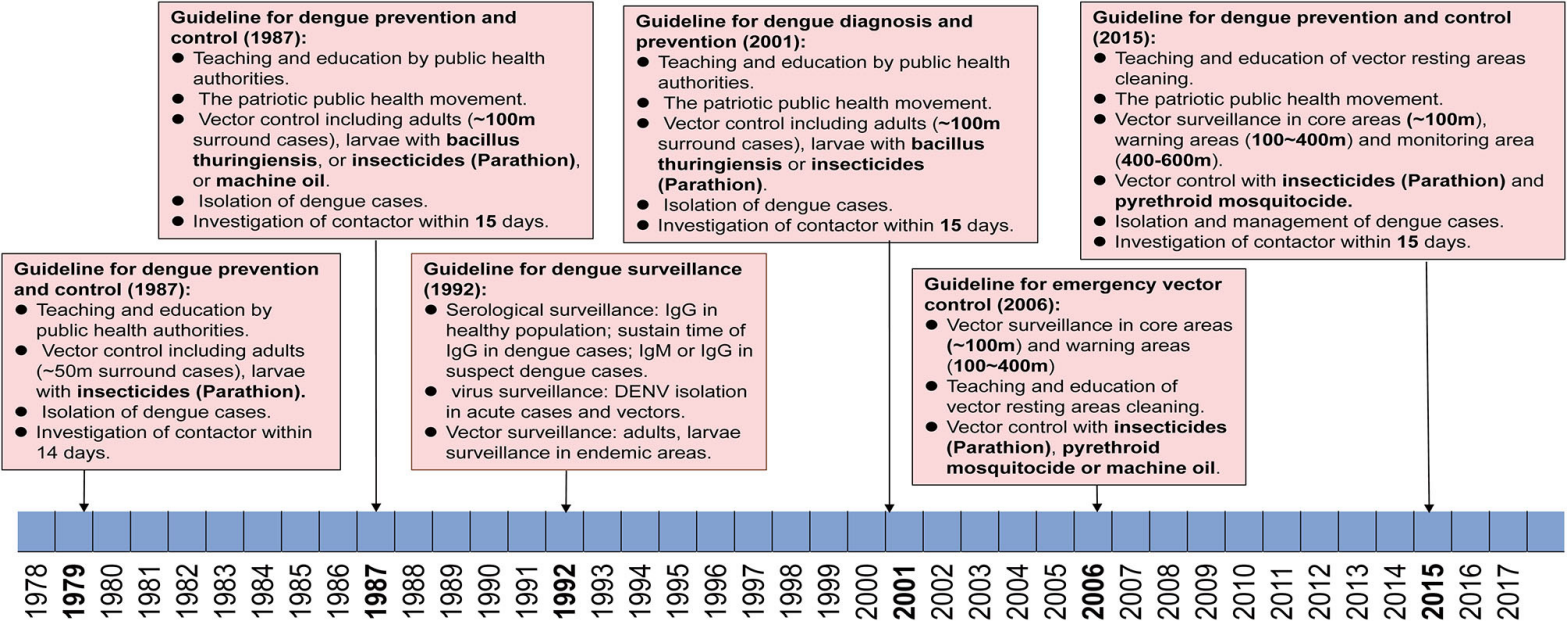
Reforming of dengue prevention and control strategies from 1979–2017. Criteria and policies' formulation and development in Guangdong are summarized according to the date of application and dengue epidemics.

There were three versions of guidelines for dengue prevention and control published formally by the Guangdong provincial public health authorities in 1979, 1987, and 2015 (Figure 5). During this period, dengue was first considered as a notifiable infectious disease, leading to the initiation of case and vector surveillance in Guangdong province in 1992. An emergency vectors surveillance guideline was launched in Guangdong regarding the dengue outbreak in 2006. Meanwhile, national criteria for dengue diagnosis and prevention were issued in 1988, 2001, and 2008 by the Chinese Ministry of Health.

### The First Stage: 1979–1987

Since the first massive dengue outbreak in Guangdong in 1978, a guideline was launched in the early 1979 and was immediately used to limit further outbreaks of dengue in Guangdong. The main strategies included (1) education, advertising to citizens to recognize the symptoms, transmission route, and competent mosquitoes of dengue through radio broadcasts, bulletin boards, and meetings, (2) vector control for adults and larvae of mosquitoes (~50 m surround cases) by using insecticides (Parathion), (3) isolation of dengue cases within the acute stage for 5 days, (4) investigation of contacts of dengue cases within 14 days.

### The Second Stage: 1988–2014

From 1985 to 1987, a large magnitude outbreaks of dengue occurred and resulted in more than 200,000 cases in Guangdong. Thus, a new guideline for dengue prevention and control was launched in 1988. Dengue cases and competent mosquitoes' surveillance were first introduced to the guidelines during the outbreaks. Particularly, the Breteau index (BI) (number of containers with mosquito larvae per 100 premises/houses checked, with 10 > BI ≥ 5 indicating low risk, 20 > BI ≥ 10 denoting moderate risk, and BI ≥ 20 indicating high risk) was first used to evaluate the status of surveillance areas. The virus genotype and rate of positive antiserum against DENV in the healthy population were also suggested to be conducted in 21 local municipal CDC (branch of provincial CDC, one in each city) of Guangdong, China. Referring to the control strategies, most of the control measures were kept as before with updating, including (1) education, advertising to citizens through radio broadcasts, television, bulletin boards, and meetings, (2) community-based patriotic public health movement to clean the resting areas of mosquitoes. (3) Vector control surrounding dengue cases (~100 m) were performed by using either insecticides (Parathion) to kill adults or *Bacillus thuringiensis* and machine oil to stop the development of larvae, (4) isolation of dengue cases within acute stage for 6 days, and implementation of vector control as well, (5) investigation of contacts of dengue cases within 15 days and isolate any suspect cases if necessary.

### The Third Stage: 2015–Now

In 2014, the third largest dengue outbreak occurred in Guangdong. In early 2015, the provincial public health authorities released a new version of the guideline for dengue prevention and laboratory diagnosis referring to new tendencies and dynamics of dengue in Guangdong. (1) Reinforced vector surveillance will be implemented all over the province with more Mosquito-Ovitraps, more frequent surveillance, and upgraded trap equipment. (2) In the event that a dengue outbreak occurs, three regions are classified as surrounding cases, including core area (~200 m), warning area (200–400 m), and monitoring area (400–600 m). The collection of BI index data should be performed within 2 days since the outbreak is reported in core areas, and repeated every three days till BI < 5. In warning areas, BI index data are collected weekly till the BI < 5. In the monitoring areas, these data are collected every 14 days till the end of this outbreak. (3) Six levels of emergency responses are defined in accordance with the guidelines (level I, BI index > 10 in one community/town, without case report, level II, BI index > 10 with imported/local cases, level III, BI index > 10, with local cases > 5 within 7 days in one community/town, level IV, BI index > 10 with local cases > 10, or > 1-fold of average number of cases within 5 years in the same area, level V, BI index > 10 with local cases > 100, or > 2-fold of average cases within 5 years in the same region or incidence of IV events in no less than two cities in Guangdong, and level VI, incidence of V events in two provinces, including Guangdong at the lowest). (4) Education of public and community-based patriotic public health movement should be combined to wipe the resting areas of mosquitoes, either in public or family areas. (5) Implementation of mosquito control strategies should be conducted intermittently with low toxic pyrethroid mosquitocide recommended by WHO, such as deltamethrin, cypermethrin, and allethrin. (6) Alert of imported dengue cases and case management will be conducted either in hospital or family, as early as possible.

## Discussion

Dengue was the most important arboviral disease in Guangdong, China from 1978 to 2017 with epidemics occurring almost annually. Outbreaks of dengue showed a seasonal distribution from July to October, which was consistent with an archived study during 1990–2018 (24), but different from dengue-endemic countries in Southeast Asia, e.g. Thailand, where about 73% of cases were reported in the rainy season from May to September (25). In Malaysia, dengue outbreaks occurred from July to March of the next year. The highest risk period was between November and March (26). The unique seasonal pattern of dengue incidence and periodic outbreaks in specific years reflects the interaction between climatic conditions including rainfall and temperature, mosquito vectors, circulating viruses, and human immunity (27, 28). In Guangdong, the dengue epidemic season was closely related to mosquito ecology. A large amount of rainfall from July to October typically increases mosquito breeding areas, which leads to an increase in the mosquito population density and thus increases the risk of dengue transmission (29). *Ae. albopictus*, a DENV vector in Guangdong, is relatively adaptable to low-temperature environments (30–32), suggesting that authorities need to conduct mosquito vector surveillance more closely.

Dengue occurred in all age group populations in the Guangdong province, but mainly in young and middle-aged adults, which showed a broader age distribution than another study in China (aged 25–34) (33), which was in agreement with the middle-age group that was the mostly affected population in Guangdong. According to the Statistics Bureau of Guangdong Province (http://stats.gd.gov.cn/), the portion of people aged 15–59 was 73.39% and 68.80%, respectively, in the 6th population census in 2010 and 7th population census in 2020. However, a study in Khon Kaen, Thailand showed that about 90% of dengue cases occurred in individuals less than 30 years old, and cases in children and early teens aged 5–14 years accounted for 51.1% among all groups (25). More than 75% of DENV infections were asymptomatic or mild, and 5% or less of patients progressed to severe DHF/DSS (34, 35). Contrarily, only a few cases of DHF/DSS have been reported in Guangdong province. The incidence of monoserotype in each year and low infection rates in the population may be a reasonable explanation (36). Indeed, most outbreaks of dengue in Guangdong were caused by the dominant serotype DENV 1 since 2001 (36). However, a serotype shift from DENV 1 to DENV 2 was going on after 2010, thus public health authorities should stay on alert for the transmission of multiple serotypes in Guangdong province, which may signal the potential for DHF/DSS incidence in the future.

A previous study showed that more than 87.5% of imported dengue cases detected at Guangzhou customs from 2009 to 2015 came from Southeast Asian countries (37). Our findings indicated that dengue cases frequently imported from Southeast Asian countries were the major source of local outbreaks in Guangdong province. The most solid evidence was the quarantine policy after COVID-19 pandemic in 2020. A strict “14 + 7” quarantine policy (14 days in quarantine stations plus 7 days quarantine at home) prevented most exported COVID-19 to China, as well as dengue. According to the report on Notifiable Infectious Diseases in 2020 released by the Guangdong Provincial Health Commission, 58 imported cases of dengue were reported in the quarantine station, while no local cases were detected, which means the incidence of dengue decreased by 99.06% compared with the same period in 2019. As of September 2021, fewer than 10 imported dengue cases were reported in Guangdong. Nevertheless, epidemiological data cannot simply conclude that dengue was not localized in Guangdong. Additional lines of evidence, e.g. virus evaluation data, are still needed to trace any potential local transmission of DENV.

Normally, DENV evolved gradually overtime on the same evolutionary branch in areas with local transmission of dengue (38). However, the genetic data of DENV in Guangdong were significantly different from those common to endemic areas. The genetic analysis of DENV 1–4 showed a complex and varied incidence of DENV transmission in Guangdong. DENV 1 and DENV 2 were the major serotypes. Most DENV 3 were imported cases, and no local transmission of DENV 4 was reported. A further time-lapse dynamic evolutionary analysis of DENV 1–4 confirmed our findings. Firstly, the combined analysis of international reference and local transmitted DENV strains indicated a global dynamic trend of dengue, the charting geographical segregation and spatial expansion of DENV. Most of the imported cases of China were from Southeast Asian countries, e.g. Thailand, Vietnam, and Singapore. Estimated time origins of DENV 1–4 were comparable (overlap in 95% HPD) to those from other archived studies with similar sequence data sets (39). For example, our previous study (20) estimated the time origin of DENV 2 to be 1882 (95% HPD: 1880–1910), which was concordant with 1898 (95% HPD, 1882–1915) in this study, and was also consistent with previously reported data (40, 41). Second, most dengue outbreaks in China could be traced to corresponding imported cases. For each outbreak, isolates belonged to independent clusters and showed no evolutionary change over time, except for a few instances where transmission persisted into the next year, e.g. 2002–2003 and 2011–2012 in Guangzhou (42). In these years, warm winters may have allowed adult vectors to persist throughout the winter months, enabling transmission when mosquitoes are typically dormant (43). Thirdly, the evolutionary characteristics of all local strains of DENV were consistent with globally transmitted strains, showing no localized branches in Guangdong, China. The most likely local transmission branch was found in the third cluster of Genotype I, DENV 1. We found that it was first imported into China in 2006 and detected several times in Guangzhou, Foshan, Zhongshan in 2009 and 2015, respectively. However, it also appeared in Singapore and Australia in 2013–2014. Li et al. suggested it was exported from China to those countries, but lacked epidemiology evidence (44). Moreover, we did not detect it anymore in those cities in Guangdong after 2015. An alternative explanation is that passive surveillance alone may have been too weak to detect local transmission of the DENV 1 genotype in question. In the future, a better coordinated active surveillance system in Guangdong involving hospitals, local CDC, and customs would improve our ability to resolve questions about the local DENV transmission.

Three editions of guidelines for dengue prevention and control were issued formally by the Guangdong provincial public health authorities, first in 1979 and then updated in 1987 and 2015, respectively. The strategies of dengue prevention and control mainly depended on education of citizens on the prevention of dengue, vector control, and isolation of dengue cases in the first version of guidelines. The second version was updated with BI as index of vector density, genotyping, and serological surveillance in all 21 local CDC of Guangdong, as well as prolonged isolation time of cases and expanded vector control strategies. The second version of guidelines was implemented for three decades until 2014, when an unexpected pandemic of dengue occurred and expanded to all 21 local cities in Guangzhou, China. Then the third version of guidelines was launched in 2015. The major update of the third version of the guidelines was on the categories of outbreak scales, for which more precise control strategies could be implemented accordingly. In addition, implementation of mosquito control was conducted intermittently with low toxic pyrethroid mosquitocide, including deltamethrin, cypermethrin, and allethrin rather than previously high toxic Parathion, which means a more environmentally and sustainable vector control strategy has been established in Guangdong, China. With the global climate changing, dengue has definitely expanded from tropical to subtropical areas. The global incidence of dengue has increased 30 times within the last five decades (10). With the implementation of China's The Belt and Road Policy, trade and personal communications between China and countries in Southeast Asia, South America, and Africa will become more frequent. Hence the threat of importation of emerging arbovirus disease will increase significantly (45), including Zika virus (ZIKV), Chikungunia virus (CHIKV), yellow fever virus (YFV), as well as DENV. Although the present quarantine policy prevents most emerging infectious diseases getting exported to China, a fever- and symptoms-based screening system in customs is still not sensitive enough to detect the most imported dengue, Zika virus disease, and yellow fever cases. A more reasonable and sustainable early detection system of imported infectious disease in customs is still needed after the end of the global pandemic of COVID-19.

In conclusion, we examined DENV transmission in Guangdong, China in-depth over the last four decades through a comprehensive analysis of demographic, epidemiology, serological assay of healthy adults, control strategies, and molecular evolution of DENV. We found that although with consecutive annual outbreaks, dengue was still not an endemic arbovirus disease in Guangdong, China. The corresponding control strategies of dengue for public health authorities will mainly focus on early detection of imported cases, precise vector control regarding explosive outbreak events, management of confirmed cases, and effective evaluation of implemented control strategies. In addition, a more sensitive screening system of imported cases in customs, vector surveillance system in communities, fast diagnosis, and management of confirmed cases maybe the advanced experiences in Guangdong, China, and probably could be an outstanding example of arbovirus disease control for west pacific areas.

## Limitations

There are a few caveats with the epidemiological data reported in this study. Firstly, dengue surveillance data during the period of 1978–1989 were obtained from local authorities' records archived in Guangdong Provincial Center for Disease Control and Prevention, whereas after 1989 dengue became a national notifiable disease in China, and dengue was diagnosed in accordance with the unified criteria issued by the Chinese Ministry of Health. This shift may be a link to the dramatic reduction in case numbers due to changes in case definition, training for a new reporting system, and slow progress or delays in implementation. Secondly, there were two updates of surveillance practices in 1990 and 2005. Dengue cases were reported monthly by provincial CDC during 1990–2004, whereas within 24 h by sentinel hospitals during 2005–2017. This retrospective surveillance from 1990–2004 may have been too weak to reveal the real-time dynamics of dengue in China. Thirdly, the data set charts changed with the reforming of policies and surveillance systems, and the detailed information of imported dengue cases before 2005 were not available, as well as molecular data in either clinic or laboratory.

## Data Availability Statement

The datasets presented in this study can be found in online repositories. The names of the repository/repositories and accession number(s) can be found in the article/Supplementary Material.

## Ethics Statement

In this study, serological surveys of healthy donors were obtained from 14 counties in Guangdong (18). The serum sampling protocol from dengue cases and healthy donors was approved by the Ethics Committee of the Guangdong CDC. All associated procedures were performed according to the ethics regulations associated with human being sampling. Patients' information was collected from local hospitals. Written informed consent was obtained from the participants or their legal guardian/next of kin to participate in this study and for the publication of any potentially identifiable images or data included in this article.

## Author Contributions

JSun: conception and design, had full access to all of the data in the study, takes responsibility for the integrity of the data, accuracy of the data analysis, and supervision. FC and LT: statistical analysis. JSun, LS, and LLin: obtained funding. All authors: acquisition or interpretation of data, critical revision of the manuscript for important intellectual content, and final approval of the version to be published.

## Funding

The National Key Research and Development Program of China (2020YFC1200104) and the Guangzhou Science and Technology Program (201904010012).

## Conflict of Interest

The authors declare that the research was conducted in the absence of any commercial or financial relationships that could be construed as a potential conflict of interest.

## Publisher's Note

All claims expressed in this article are solely those of the authors and do not necessarily represent those of their affiliated organizations, or those of the publisher, the editors and the reviewers. Any product that may be evaluated in this article, or claim that may be made by its manufacturer, is not guaranteed or endorsed by the publisher.

## Supplementary Material

Supplementary Figure 1 Epidemic season and geographic distribution of dengue in Guangdong from 1978 to 1989. **(A)** The number of cases in each month is presented by the average number in each month within the previous 12 years. The incidence peak is marked by dark blue. **(B)** Geographic distribution of accumulated dengue cases from 1978 to 1989 in 21 cities of Guangdong. The number of dengue cases in each city is shaded according to the logarithmic scale (log10).

Supplementary Figure 2 Annual distribution of dengue between 1990 and 2017 **(A)**. Meta-analysis of yearly peak period found the overall peak time was September-October, with medium heterogeneity (I2 = 67.90%, *p* < 0.01) **(B)**.

Supplementary Figure 3 Bayesian evolutionary tree of DENV 1 genotype I. Source countries/regions of 9 clusters of DENV 1 Genotype I are presented by colors. The red arrow marks suspected local transmission branches. The vertical dashed line indicates the recent expansion of genotype I from Thailand.

Supplementary Figure 4 Bayesian evolutionary tree of suspected local evolutionary branch in DENV 1 genotype I. Source countries/regions of the strains of DENV 1 genotype I are presented by different colors.

Supplementary Figure 5 Bayesian evolutionary tree of DENV 1 genotype IV. Source countries/regions of 3 clusters of DENV 1 genotype IV are presented by colors.

Supplementary Figure 6 Bayesian evolutionary tree of DENV 1 genotype V. Source countries/regions of 3 clusters of DENV 1 genotype IV are presented by colors.

Supplementary Figure 7 Bayesian evolutionary tree of DENV 3. Source countries/regions of each strain and different genotypes of DENV 3 are presented by colors. Red dots represent imported cases, blue dots represent local cases, and brown dots are internal branch points with posterior probability = 1.0.

Supplementary Figure 8 Bayesian evolutionary tree of DENV 4. Source countries/regions of each strain and different genotypes of DENV 4 are presented by colors. Red dots represent imported cases, blue dots represent local cases, and brown dots are internal branch points with posterior probability = 1.0.
