# Supplementary figures and images for: Dengue and Dengue Virus in Guangdong, China, 1978–2017: Epidemiology, Seroprevalence, Evolution, and Policies
Source: Front Med (Lausanne). 2022 Mar 21;9:797674. doi: 10.3389/fmed.2022.797674 (PMC8979027; doi:10.3389/fmed.2022.797674)

A

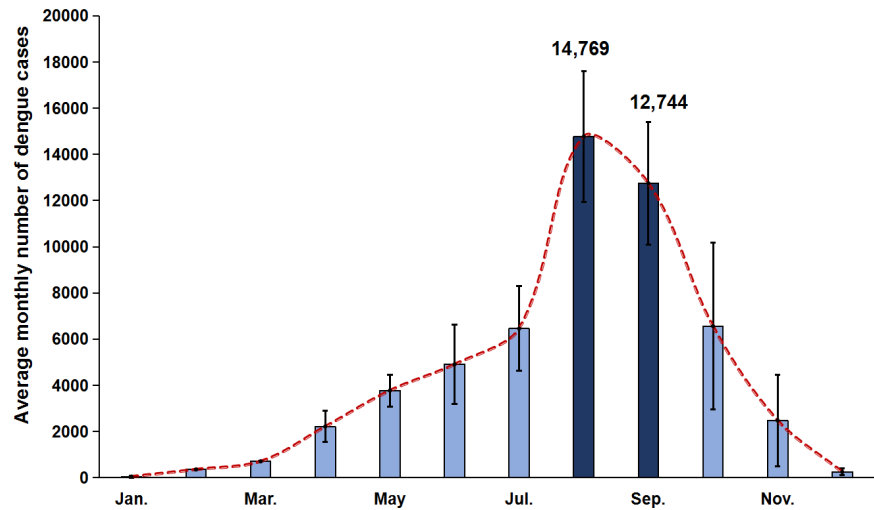

B

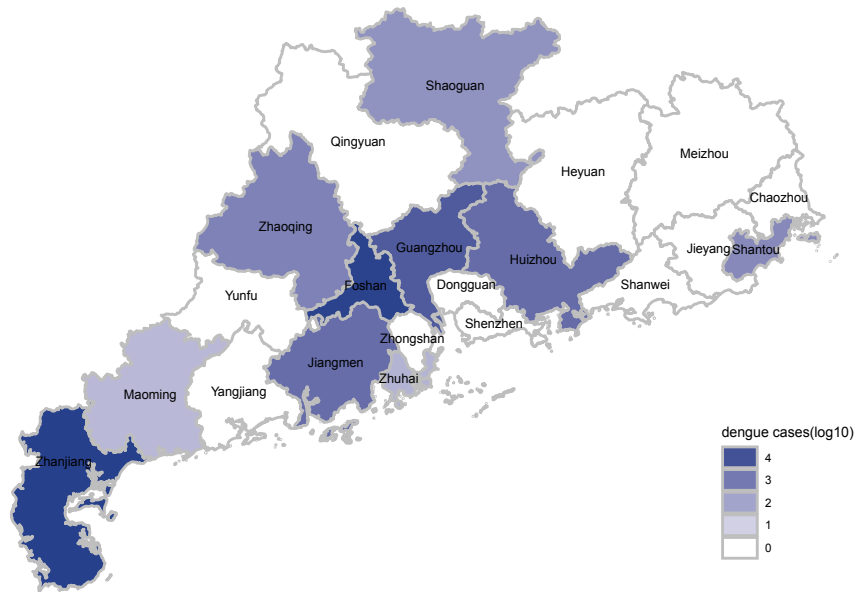

Supplement: Supplementary Figure 1 — Epidemic season and geographic distribution of dengue in Guangdong from 1978 to 1989. (A) The number of cases in each month is presented by the average number in each month within the previous 12 years. The incidence peak is marked by dark blue. (B) Geographic distribution of accumulated dengue cases from 1978 to 1989 in 21 cities of Guangdong. The number of dengue cases in each city is shaded according to the logarithmic scale (log10). [file Data_Sheet_1.PDF]

A

Dengue cases

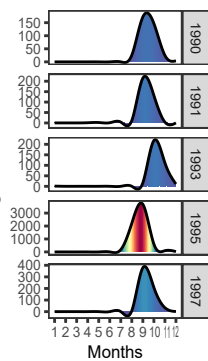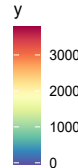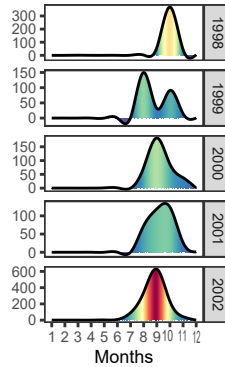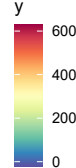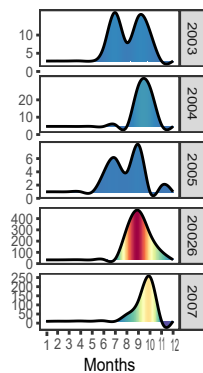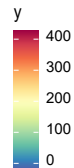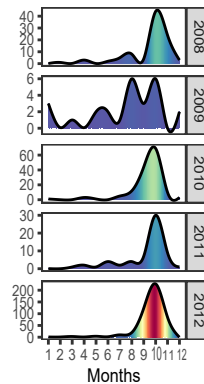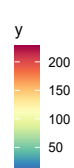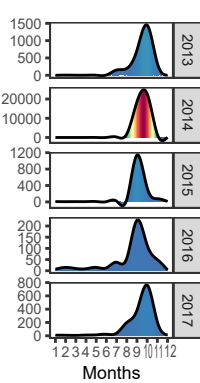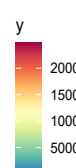

1990  
1991  
1993  
1994  
1995  
1997  
1998  
1999  
2000  
2001  
2002  
2003  
2004  
2005  
2006  
2007  
2008  
2009  
2010  
2011  
2012  
2013  
2014  
2015  
2016  
2017

B

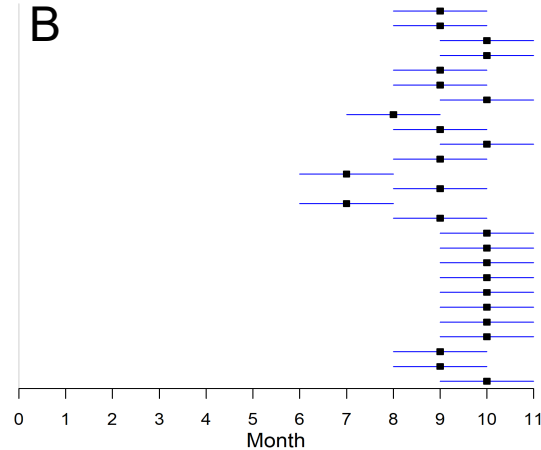

Supplement: Supplementary Figure 2 — Annual distribution of dengue between 1990 and 2017 (A). Meta-analysis of yearly peak period found the overall peak time was September-October, with medium heterogeneity (I2 = 67.90%, p < 0.01) (B). [file Data_Sheet_2.PDF]

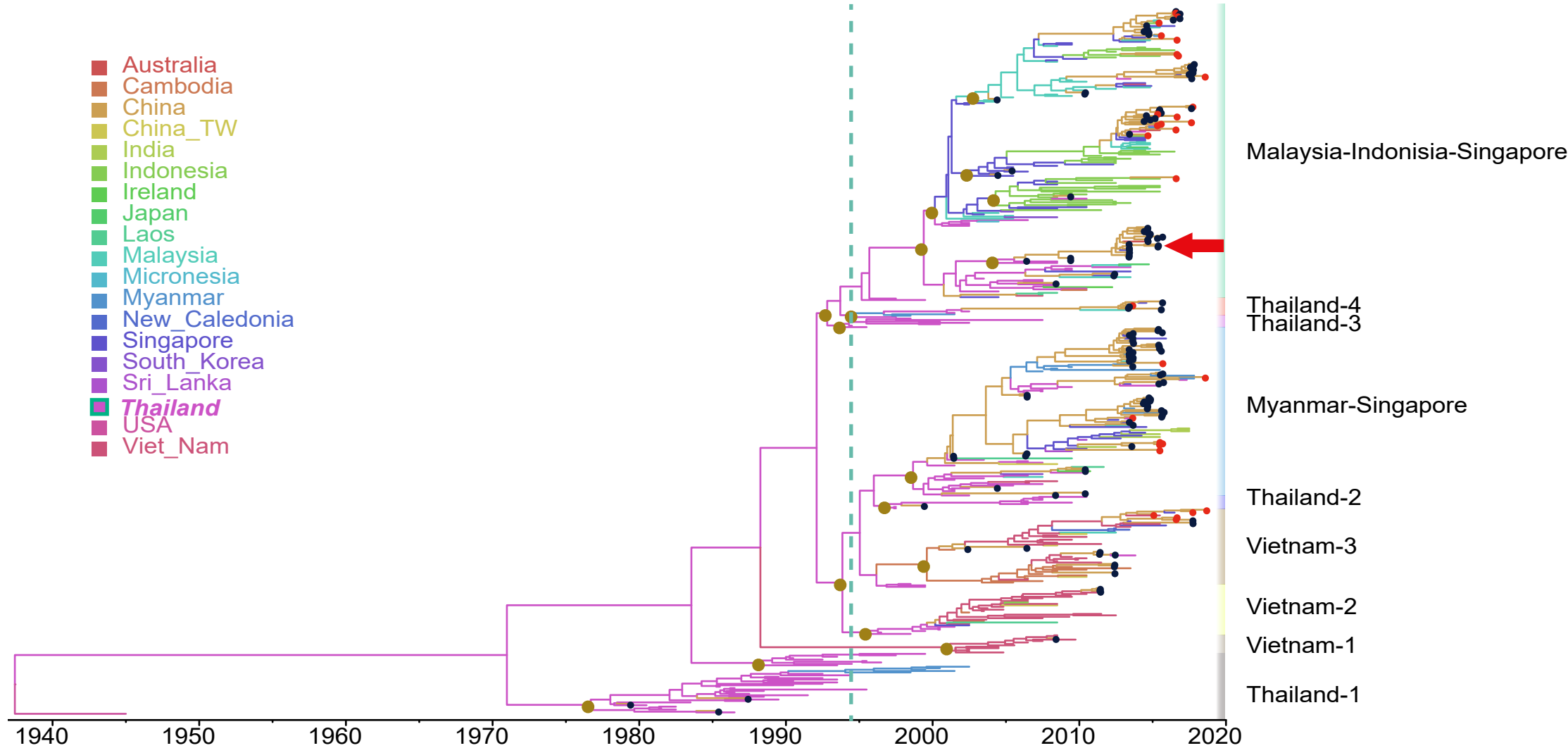

Supplement: Supplementary Figure 3 — Bayesian evolutionary tree of DENV 1 genotype I. Source countries/regions of 9 clusters of DENV 1 Genotype I are presented by colors. The red arrow marks suspected local transmission branches. The vertical dashed line indicates the recent expansion of genotype I from Thailand. [file Data_Sheet_3.PDF]

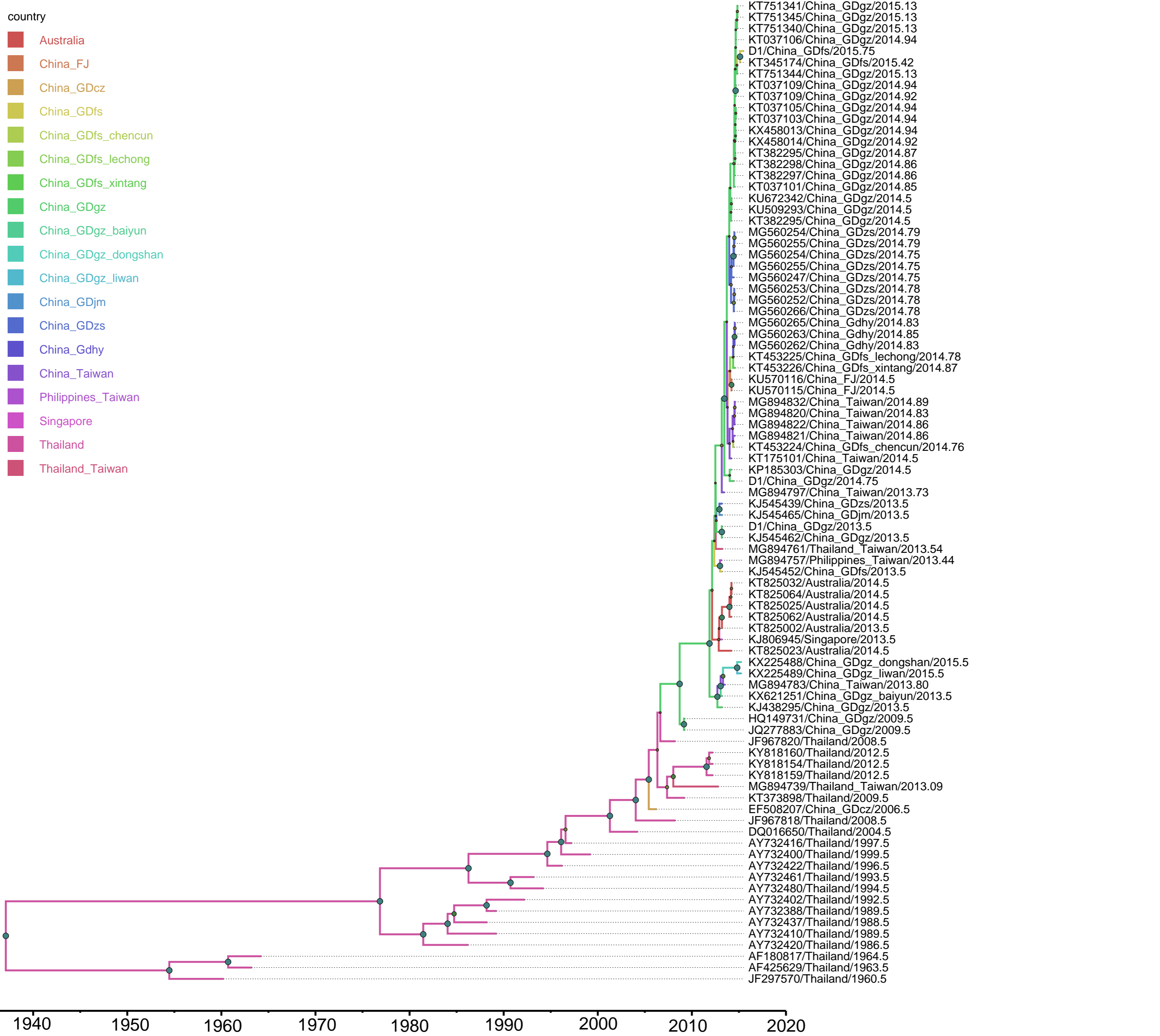

Supplement: Supplementary Figure 4 — Bayesian evolutionary tree of suspected local evolutionary branch in DENV 1 genotype I. Source countries/regions of the strains of DENV 1 genotype I are presented by different colors. [file Data_Sheet_4.PDF]

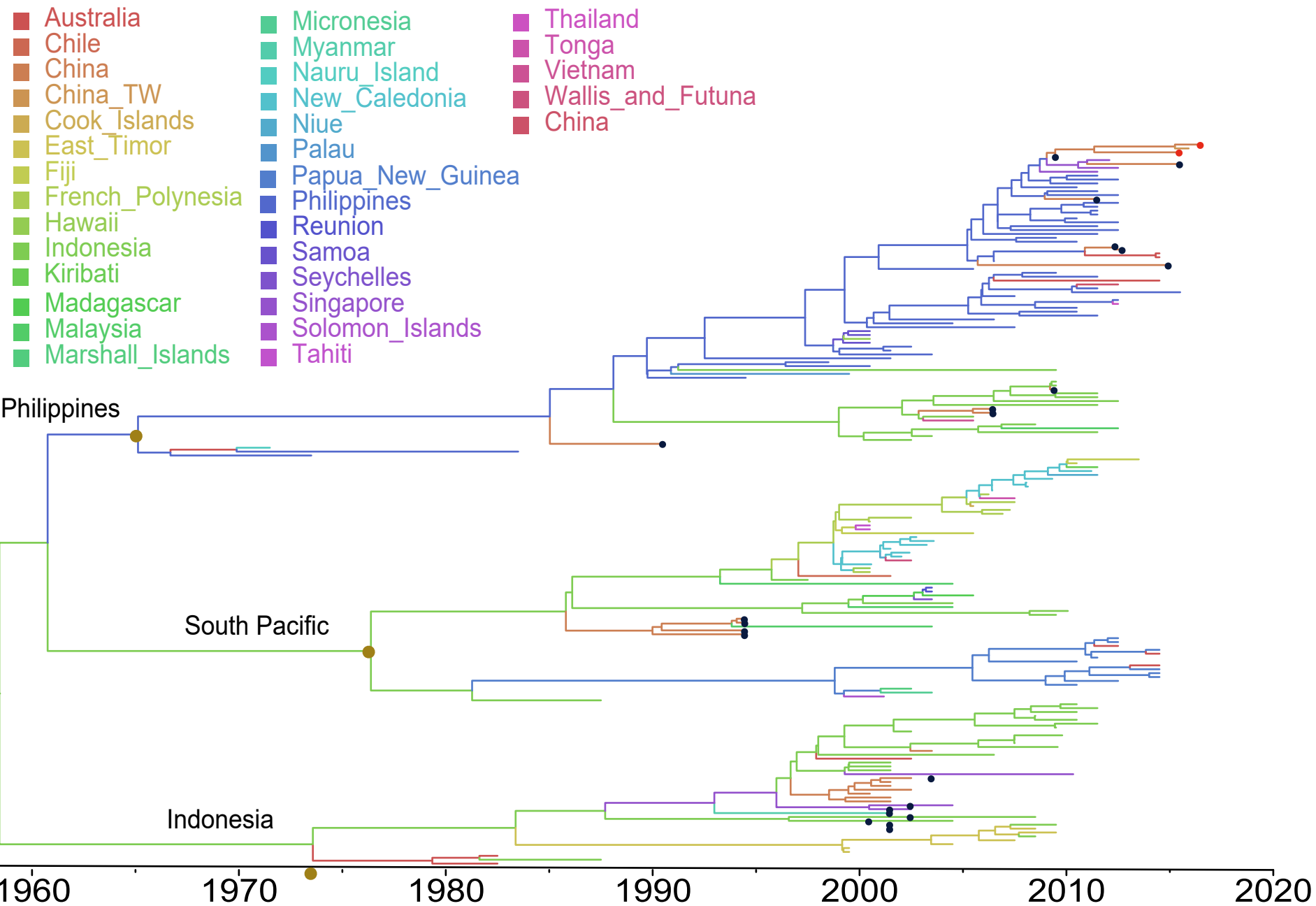

Supplement: Supplementary Figure 5 — Bayesian evolutionary tree of DENV 1 genotype IV. Source countries/regions of 3 clusters of DENV 1 genotype IV are presented by colors. [file Data_Sheet_5.PDF]

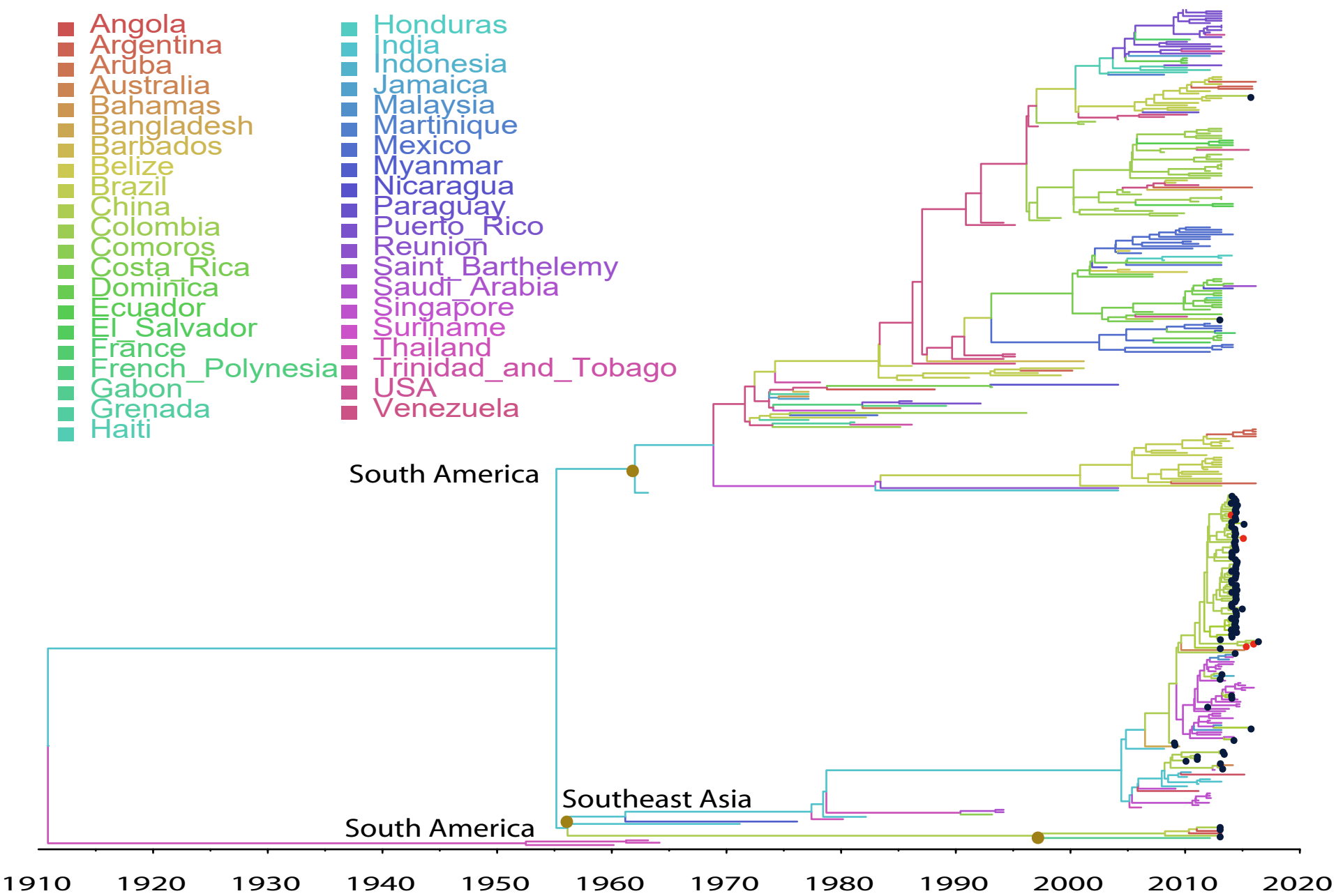

Supplement: Supplementary Figure 6 — Bayesian evolutionary tree of DENV 1 genotype V. Source countries/regions of 3 clusters of DENV 1 genotype IV are presented by colors. [file Data_Sheet_6.PDF]

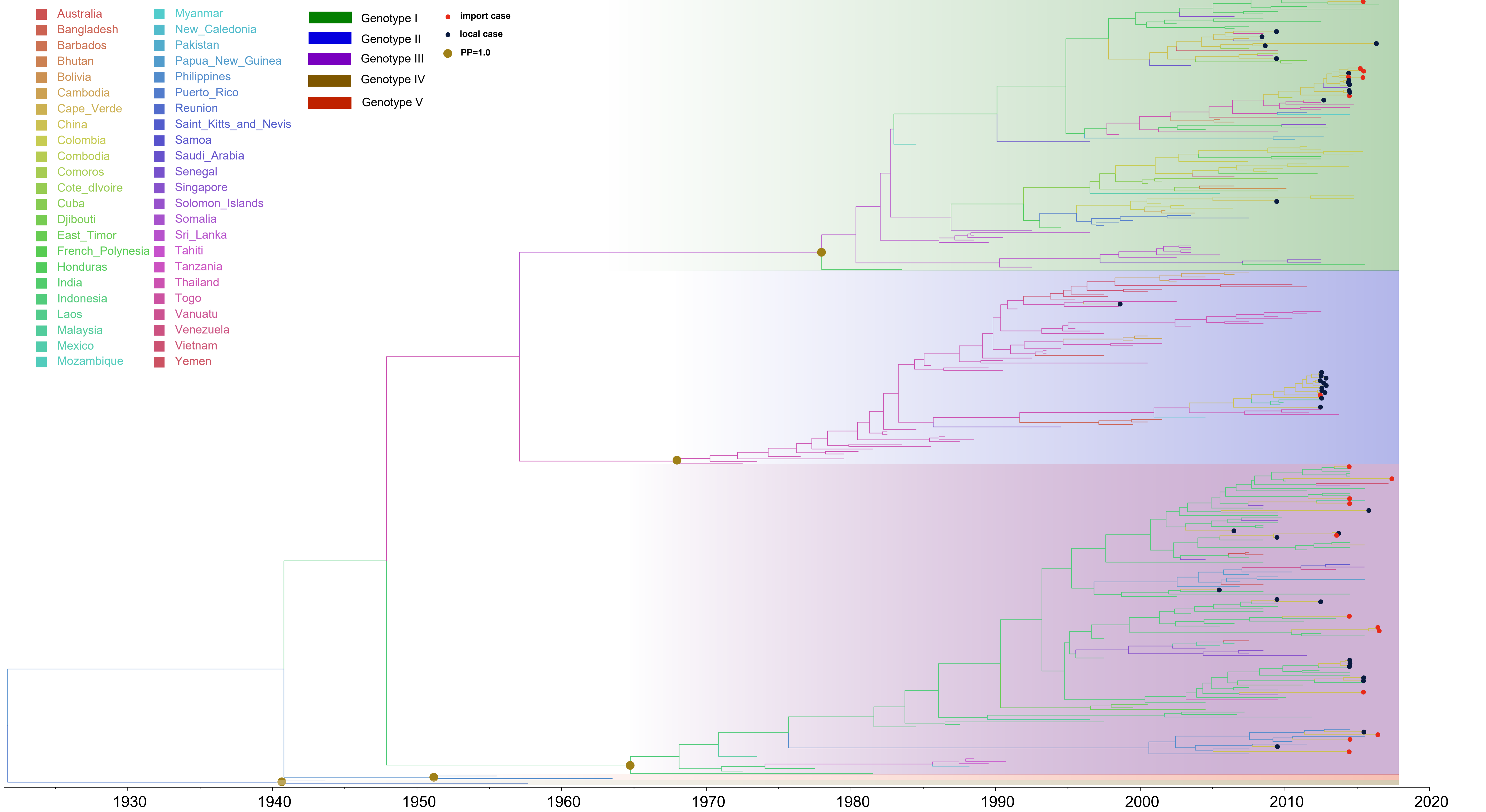

Supplement: Supplementary Figure 7 — Bayesian evolutionary tree of DENV 3. Source countries/regions of each strain and different genotypes of DENV 3 are presented by colors. Red dots represent imported cases, blue dots represent local cases, and brown dots are internal branch points with posterior probability = 1.0. [file Data_Sheet_7.PDF]

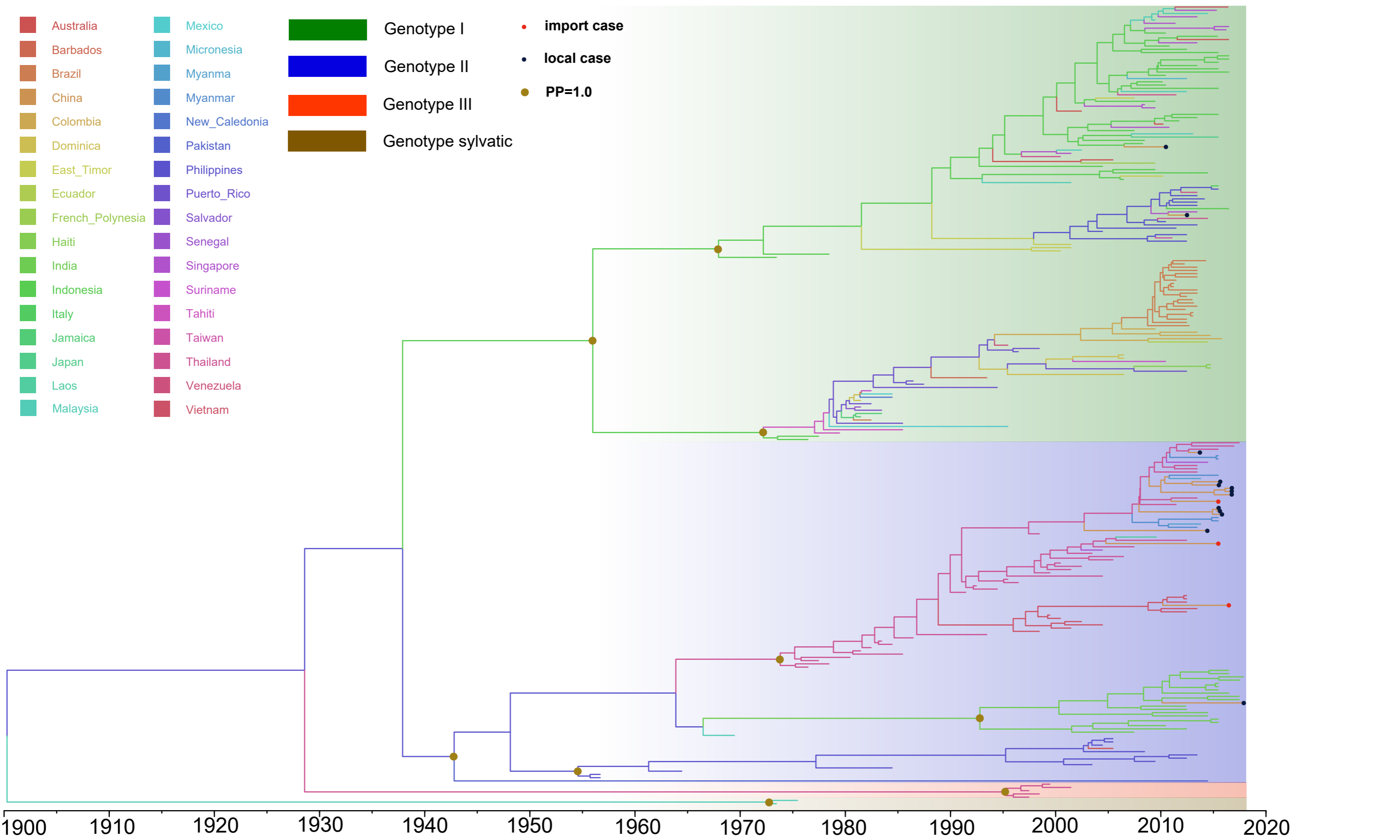

Supplement: Supplementary Figure 8 — Bayesian evolutionary tree of DENV 4. Source countries/regions of each strain and different genotypes of DENV 4 are presented by colors. Red dots represent imported cases, blue dots represent local cases, and brown dots are internal branch points with posterior probability = 1.0. [file Data_Sheet_8.PDF]
